# Attention Wins over Sensory Attenuation in a Sound Detection Task

**DOI:** 10.1371/journal.pone.0136585

**Published:** 2015-08-24

**Authors:** Liyu Cao, Joachim Gross

**Affiliations:** University of Glasgow, Glasgow, United Kingdom; Birkbeck College, UNITED KINGDOM

## Abstract

‘Sensory attenuation’, i.e., reduced neural responses to self-induced compared to externally generated stimuli, is a well-established phenomenon. However, very few studies directly compared sensory attenuation with attention effect, which leads to increased neural responses. In this study, we brought sensory attenuation and attention together in a behavioural auditory detection task, where both effects were quantitatively measured and compared. The classic auditory attention effect of facilitating detection performance was replicated. When attention and sensory attenuation were both present, attentional facilitation decreased but remained significant. The results are discussed in the light of current theories of sensory attenuation.

## Introduction

Sensory consequences of self-initiated actions are perceived weaker and lead to weaker brain responses compared to passively received sensory stimuli (sensory attenuation, SA). Sato [1] reported that a tone generated by pressing a button has a lower perceived loudness compared to a tone generated by computer. SA has also been reported in the visual and somatosensory domain [2, 3]. Single cell recordings from animal studies [4, 5] and human neuroimaging studies [6, 7] confirmed that there is an attenuated response in primary sensory cortex after a motor response. A forward model was proposed to account for SA [8]. It was suggested that before or during movement an efference copy [9] of motor commands leads to the generation of a corollary discharge [10] in the corresponding sensory areas, thus enabling participants to predict the imminent stimulus. If the prediction is in accordance with the incoming stimulus, SA will be observed [11].

SA may be important for individuals in many different ways, such as in keeping sense of agency or speech monitoring [12–15]. However, there are also situations where detecting an outcome from self movement is crucial. For example, when walking on a frozen lake, subjects must be paying full attention to a possible ice crack sound so that it can be detected instantly. But the existence of SA seems to be disadvantageous as it lowers sensitivity to the sound. This raises the interesting question how SA and attention differentially affect stimulus processing. Attention is considered as a cognitive ability to facilitate sensory processing. In general, behavioural performance and electrophysiological brain responses are enhanced for attended as compared to unattended sensory stimuli [16–18]. For example, Greenberg and Larkin [16] investigated how attending to a particular frequency tone could improve its detection. They found a significant increase of detection rate for attended tone compared to unattended tone, which should be equally detectable without the intervention of attention. Since SA and attention have opposing effects on sensory processing, it is an interesting question what the result would be if both coexist. Several electrical neuroimaging studies have explored this question showing independent or interdependent relationship between the two (e.g., ref. [19]). According to a framework proposed by Schröger, Marzecova and SanMiguel [20], attention acts as a voluntary gain control, which can modulate the amplitude of brain responses to a stimulus. Depending on the predictability, a stimulus can receive different levels of attentional gain control. Thus there is a close relationship between attention and SA/prediction. Many of the studies manipulated attention and then compared SA effects at different attention levels. For example, Timm et al. [19] found that SA effects didn’t change at different attention levels. However, the net effect when both attention and SA coexist is largely ignored and poorly understood. We studied this question using a behavioural auditory detection task. In the task, participants were required to detect a near-threshold sound, where attention and SA were manipulated at the same time. With this design we can measure the effect of attention and SA at the same time thus enabling a direct comparison between the two.

## Methods

### Participants

28 participants (13 females; mean age = 25.1, SD = 7.0; two left handed) were recruited through poster advertising on campus. They gave written informed consent prior to the experiment and received £6/hour compensation. The experiment was conducted conforming to the ethical codes of the declaration of Helsinki and was approved by Ethics Committee of College of Science & Engineering, University of Glasgow.

### Main task

**Fig 1:**
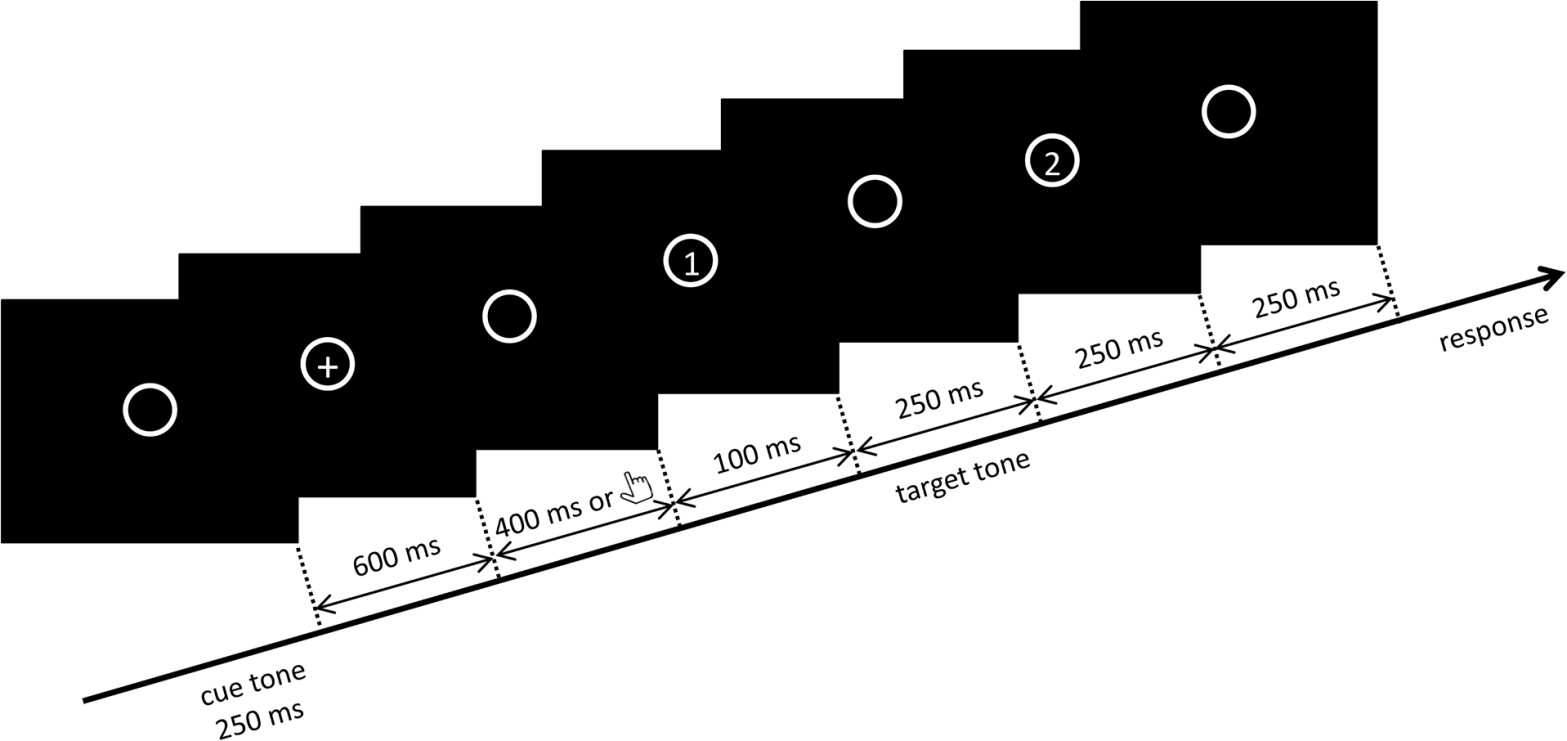
Timeline of one trial in the main task. Participants first heard a cue tone. 600 ms later, a cross appeared in the centre of the screen and then the sound detection task started either following participants’ button press or after a delay of 400 ms. The inter-trial interval was 1000 ms. In this example, the target tone is in the first interval.

An auditory attention paradigm adapted from Borra, Versnel (21) was used (Fig 1). In the task, participants were required to detect a near-threshold target tone. In the beginning of a trial, a cue tone (clearly audible) was first played. After 600 ms, a cross appeared in the centre of the screen. In ‘no SA’ condition, the cross was present for 400 ms, and a sound detection task started automatically 100 ms after its offset. In the sound detection task, a near threshold target tone was always presented in either the first or the second interval, which participants were required to make judgment of. In ‘SA’ condition, participants were told to press a button (number ‘2’ on numeric section of a standard keyboard) with right index finger when they saw the cross. The same detection task started 100 ms after the button press, which was supposed to activate the forward model and lead to SA effect. The cue tone was always 1000 Hz, and the target tones could be 880 Hz, 1000 Hz, or 1120 Hz. Participants were told to ‘search for’ the same target tone in the sound detection task as the cue tone, whereas being informed that the target tone could be different from the cue tone at the same time. Thus, in the sound detection task, participants’ attention was drawn to the 1000 Hz tone (‘attended’), but not to the 880 Hz or 1120 Hz tone (‘unattended’). Previously, an auditory attention effect has been reported and is reflected in improved detection performance for attended target tone compared to unattended target tone [16, 21]. We expect to replicate this in ‘no SA’ condition, which is similar to a standard auditory attention task.

All tones had durations of 250 ms (8 ms rise/fall). Continuous background white noise (volume set to a comfortable level) was presented during the experiment to control for environmental noise. The cue tone was 12 dB lower than the background noise and it was clearly audible. The volume of target tones was determined through a pre-test phase (see below). Auditory stimuli were delivered through headphones (Model: Sony MDR-XD100).

Each condition consisted of 128 trials and was divided into two blocks. In half of trials, the target tone was 1000 Hz; in the other half of trials, the target tone was 880 Hz or 1120 Hz (each 50%). Probability of target tone presentation was equally distributed across the two intervals (both intervals had the duration of 250 ms, which is the same as the duration of the tone). The correspondence between response buttons and intervals was counterbalanced among participants (half participants pressed ‘A’ for the first interval, ‘D’ for the second interval; the other half pressed ‘A’ for the second interval, ‘D’ for the first interval). The order of the two conditions was also counterbalanced. Participants were instructed to avoid any unnecessary movement during the experiment.

### Pre-test

An approximate detection threshold for each participant was established before the experiment. The task was similar to the main task, but no cue tone was played. Five 1000Hz tones with different intensities (normally from -30.5 dB to -20.5 dB with 2.5 dB increment and the intensity was compared to background noise) were each presented across 48 trials in a random order. Then the accuracy data for the five tones were fitted with a sigmoid function and 85% detection threshold for the 1000 Hz target tone was determined [21]. The 85% threshold for the 880 Hz/1120 Hz target tone was attained by subtracting/adding 0.24 dB to the threshold for the 1000 Hz target tone according to the function between detection threshold and frequency reported by Green et al. [22].

### Data analysis

Corrected d′ as a measure of sensitivity between the two intervals (see formula 1 below) and response bias c as a measure of possible bias for certain responses (see formula 2 below) were calculated, respectively [23]. The data for 880 Hz and 1120 Hz target tones were concatenated because both tones were unattended. After concatenation, there were equal numbers of trials for attended (1000 Hz) and unattended tones (880 Hz and 1120 Hz). There were two factors in this study: tones could be ‘attended’ or ‘unattended’; ‘SA’ (with a button press) or ‘no SA’ (without a button press) was present in the sound detection task. Thus a within-subject 2 by 2 ANOVA was run with SPSS 19. Data from two participants were excluded due to chance level performance for all the target tones. Note that results do not change qualitatively even if data from these two participants are included.

d′=z(hitrate)−z(falsealarmrate) (1)

c=−0.5x[z(hitrate)+z(falsealarmrate)] (2)

Hitrate=(numberofhitsforfirstinterval+0.5)/(numberoftrialswithtargettoneinfirstinterval+1)

Falsealarmrate=(numberoffalsealarmsforfirstinterval+0.5)/(numberoftrialswithtargettoneinsecondinterval+1)

## Results

**Fig 2:**
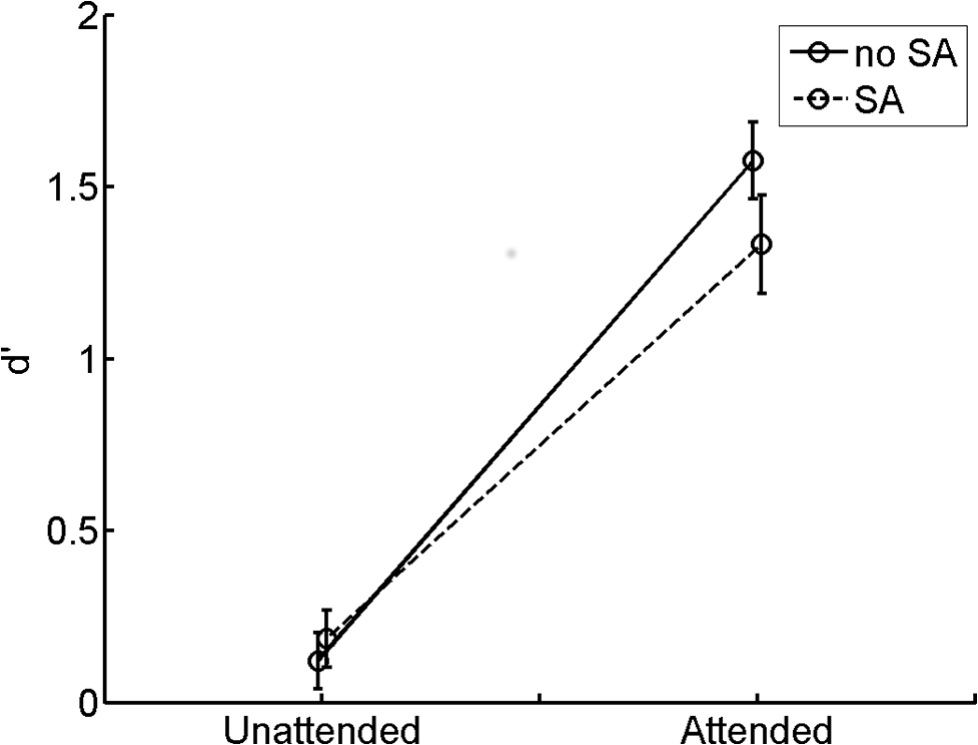
d′ results. d′ of behavioural performance for different conditions. Vertical bars stand for standard error. SA: sensory attenuation.

A 2 (‘attended’ vs ‘unattended’) by 2 (‘SA’ vs ‘no SA’) ANOVA on d′ data revealed a main effect of attention (*F*(1,25) = 94.99, *p* < .01, η^2^ = .79) and an interaction between attention and SA (*F*(1,25) = 9.89, *p* < .01, η^2^ = .28). Sensitivity for the attended tone (mean = 1.45, SD = 0.62) was larger than the unattended (mean = 0.15, SD = 0.36), which is a replication of the classic auditory attention effect (Fig 2). Post-hoc analysis for the interaction effect suggested that sensitivity for attended tone in ‘SA’ condition (mean = 1.33, SD = 0.73; mean hit rate: 71.45%, false alarm rate: 25.87%) was smaller than in ‘no SA’ condition (mean = 1.58, SD = 0.57; mean hit rate: 77.86%, mean false alarm rate: 24.59%) (*t*(25) = -2.86, *p* < .01). Attention effect still remained significant in ‘SA’ condition, so in this condition the performance for ‘attended’ tone was better than the ‘unattended’ tone (*t*(25) = 8.01, *p* < .01). There was no significant change for unattended tones between ‘no SA’ (mean = 0.12, SD = 0.42; mean hit rate: 51.52%, false alarm rate: 47.09%) and ‘SA’ condition (mean = 0.19, SD = 0.42; mean hit rate: 50.93%, false alarm rate: 43.82%)) (*t*(25) = -0.77, *p* = .45). Performance for unattended tones was at chance level and is therefore not further analysed or discussed. The main effect of SA was not significant (*F*(1,25) = 1.73, *p* = .20, η^2^ = .07). The effect size for attention and SA effect in ‘attended’ condition was 2.02 and 0.56, respectively (Cohen’s d).

Response bias c was also subjected to ANOVA analysis, and no significant results were found indicating that differences between conditions were not due to changes in response bias (see Table 1).

**Table 1:** Response bias c for different conditions (with standard deviation in brackets). There are no significant main effects (attention: *F*(1,25) = 0.72, *p* = 0.41); sensory attenuation: *F*(1,25) = 2.33, *p* = 0.14) or interaction effect (F(1, 25) = 0.84, p = 0.37).

	Attended	Unattended
SA	0.05(0.25)	0.07(0.26)
no SA	-0.05(0.31)	0.02(0.32)

## Conclusions and Discussion

We investigated the effects of SA and attention on auditory detection performance. The classic auditory attention effect was replicated, i.e., performance for the attended tone was better compared to the unattended tone. More interestingly, we found that in the presence of both SA and attention, auditory attention effect was significantly reduced but still maintained. Analysis of response bias confirmed that this result was not confounded by condition specific changes of response bias.

The predominant account for SA posits that SA results from correct prediction (with a motor origin through forward model) of incoming sensory stimuli. To build up this prediction, some studies even included a pre-test phase where participants learned the association between the motor response and sensory stimuli (e.g., Sato [1], Cardoso-Leite et al. [3]). But this is not the case in our study. There is no motor prediction due to the design of our experiment, i.e., motor response does not necessarily produce a particular tone at a particular time. The data are consistent with the idea of a general suppressive effect of motor response to auditory cortex (maybe somatosensory cortex as well, see Walsh and Haggard [24]) regardless of prediction [25, 26]. A recent animal study also found indistinguishable modulatory effect on auditory cortex across many different movement patterns [27]. This general suppression may be developed from daily experience that a motor response is always, if not all the time, associated with some kind of auditory feedback. Further studies are needed to confirm this hypothesis. Note that the general suppression effect is not mutually exclusive to motor prediction based SA, which may require some learning for association between motor response and corresponding sensory effect. Both of them may be developed from experience, but intensive learning during a short period of time makes motor prediction based SA salient over the general suppression effect.

When the stimuli were attended, a clear SA effect was still observed, which is consistent with recent brain imaging studies [19, 20]. An alternative explanation for the SA effect is that the decreased performance might result from attentional withdrawal from the discrimination task caused by the button press. As with conventional sensory attenuation studies, this is difficult to address experimentally because a motor response (button press) is a prerequisite for sensory attenuation, which can always be linked to attentional changes. In sensory attenuation literature, the attentional withdrawal question has already been addressed and the conclusion is that attentional withdrawal cannot explain sensory attenuation exclusively [19, 28]. For our data, an additional analysis was performed to indirectly address this question. We used coefficient variation (standard deviation divided by mean) of reaction time to the cross as a measure of attention allocated to the button press task. A small coefficient variation indicates a large attentional withdrawal from the discrimination task caused by the button press, thus should lead to a large performance decrease (SA). Therefore, if the reported effects were compatible with the attentional withdrawal account we would expect a negative correlation between coefficient variation and SA. However, the correlation is positive and not statistically significant (Spearman’s rho = 0.37, *p* > 0.05). Thus attentional withdrawal due to button press is unlikely to explain the SA.

The attention effect (Cohen’s d = 2.02) was much stronger than SA effect (Cohen’s d = 0.56) with the current paradigm. The net effect on performance with attention and SA coexisting was still above chance level, i.e., attention outperformed SA. It is important to note that the absence of SA effect when the stimuli were not attended was due to the floor effect for the unattended tone, i.e., the detection performance for unattended tone was always at chance level. In the current study, there are not enough levels of attention to study the interaction between SA and attention, which might be an interesting topic for further behavioural exploration. An interesting question would be if SA can be completely suppressed with very strong attention, or if attention can be completely suppressed with very strong SA. With the current study paradigm, the unpredictability of the stimulus shouldn’t have an effect on the attentional gain control process [20], thus we predict that a very strong attention effect has the potential to completely supress SA and a very weak attention effect can be suppressed by SA. Van Hulle and colleagues [29] found that tactile suppression was decreased if attention was focused on the to be stimulated body part, thus suggesting that attention could cancel tactile suppression. Timm et al. [19] reported an ERP study suggesting that SA is independent from attention. They compared SA effect in different attentional contexts and found that SA effect did not change in different attention conditions. While they did not report whether attention effect was still significant during SA, visual inspection of their results suggests that this is the case, which is similar to our finding. It is also interesting to compare our results with recent work on the interaction between attention and higher level prediction [30]. Higher level prediction has similar effect to SA in reducing sensory signals, but that can be reversed by attention. In our study, SA was still significant in the presence of attention, which suggests that SA is different from higher level prediction [28]. Back to the ice crack example given in introduction, it seems that SA sometimes can have adverse consequences. However, we would be very cautious in drawing this conclusion as the attention manipulated here may not be comparable to the attention paid when walking on a frozen lake.
